# The Dutch Consumer Quality Index: an example of stakeholder involvement in indicator development

**DOI:** 10.1186/1472-6963-10-88

**Published:** 2010-04-06

**Authors:** Diana MJ Delnoij, Jany JDJM Rademakers, Peter P Groenewegen

**Affiliations:** 1 Centre for Consumer Experience in Healthcare (Centrum Klantervaring Zorg). PO Box 1568, 3500 BN Utrecht, the Netherlands; 2 TRANZO (Scientific Centre for Care and Welfare), Faculty of Social and Behavioural Sciences, Tilburg University, PO Box 90153, 5000 LE Tilburg, the Netherlands; 3 NIVEL (Netherlands Institute for Health Services Research), PO Box 1568, 3500 BN Utrecht, the Netherlands; 4 Utrecht University, Department of Human Geography, Department of Sociology, PO Box 90115, 3508 TC Utrecht, the Netherlands

## Abstract

**Background:**

Like in several other Western countries, in the Dutch health care system regulated competition has been introduced. In order to make this work, comparable information is required about the performance of health care providers in terms of effectiveness, safety and patient experiences. Without further coordination, external actors will all try to force health care providers to be transparent. For health care providers this might result in a situation in which they have to deliver data for several sets of indicators, defined by different actors. Therefore, in the Netherlands an effort is made to define national sets of performance indicators and related measuring instruments. In this article, the following questions are addressed, using patient experiences as an example:

- When and how are stakeholders involved in the development of indicators and instruments that measure the patients' experiences with health care providers?

- Does this involvement lead to indicators and instruments that match stakeholders' information needs?

**Discussion:**

The Dutch experiences show that it is possible to implement national indicator sets and to reach consensus about what needs to be measured. Preliminary evaluations show that for health care providers and health insurers the benefits of standardization outweigh the possible loss of tailor-made information. However, it has also become clear that particular attention should be given to the participation of patient/consumer organisations.

**Summary:**

Stakeholder involvement is complex and time-consuming. However, it is the only way to balance the information needs of all the parties that ask for and benefit from transparency, without frustrating the health care system.

## Background

### Introduction

Several other Western countries, for example the United States, look at the Dutch healthcare system as an example [1,2]. In the Netherlands, regulated competition has been introduced in healthcare. In order to make this competition work, transparency is required [3]. Consumers and health insurers or other purchasing agencies need comparable information about the performance of health care providers in terms of effectiveness, safety and patient experiences. Apart from that, transparency is also required from the point of view of public accountability [4,5].

In centralised, state-oriented systems like the English National Health Service (NHS), government or government agencies impose national sets of performance indicators with which health care providers have to comply. The English Care Quality Commission asks "all NHS organisations to assess their performance against the Government's 24 core Standards for Better Health" [6]. However, in pluralistic systems like the United States, or in social insurance systems like the Netherlands, quality standards are not likely to be imposed by government, and the definition of indicator sets requires much more coordination and negotiation among the actors in the health care system.

In the United States, standardized indicator sets are developed by private not-for-profit organizations, such as the Joint Commission [7] and the National Committee for Quality Assurance (NCQA). The NCQA, for example, aims to build consensus among large employers, policymakers, medical professionals, patients and health insurers to decide how to measure important aspects of quality of care. The NCQA is responsible for the management of the Healthcare Effectiveness Data and Information Set (HEDIS), a set of standardized performance indicators. HEDIS is used by more than 90% of health plans throughout the United States [8]. According to the American NCQA, consensus about indicators and measurement instruments is necessary, because transformation of the health care system requires the collective will and resources of all the actors involved: employers (in the American context), policymakers, professionals, patients and health insurers [9].

In the Dutch system, private, mostly not-for-profit organisations and self-employed private practitioners, provide health care. In a system of regulated markets, these organisations and practitioners are theoretically assumed to compete with each other for health insurance contracts and for individual patients. As such, they have an incentive to advertise and to give potential consumers information about specific products or services. However, market forces do not generate comparative information without external pressure. In the Netherlands, this external pressure can theoretically be exercised by several actors: the Ministry of Health, the Inspectorate for Health Care, the Dutch Healthcare Authority ("Nederlandse Zorgautoriteit"), health insurers, and/or patient/consumer organisations.

Without further coordination, all these external actors will try to force health care providers to be transparent. For health care providers this might result in a situation in which they have to deliver data for several sets of indicators, defined by different actors. To avoid this situation, the main challenge for the Dutch health care system currently is to stimulate health care providers to be transparent about those indicators that are relevant for the Inspectorate for Health Care, health insurers and patients/consumers alike. However, at the same time the amount of data and the number of indicators that providers have to deliver, should be limited.

In the Netherlands, the performance of health care providers is measured in terms of effectiveness and safety (in the Dutch context these are called 'professional indicators'), and patient experiences with quality aspects such as access, timeliness, information and communication, respectful treatment etc. A strong effort is made to define national sets of performance indicators and related measuring instruments. In the Dutch setting, these national indicator sets are agreed upon by all the stakeholders involved in the transparency debate. This should decrease the administrative burden on health care providers. Collecting information on performance indicators is time-consuming, costly and generally requires the cooperation of health care providers. However, by using national indicator sets, information has to be collected once and can then be used for multiple purposes.

Because of the desire to define indicator sets, not from the perspective of one dominant actor (such as the Government in England), but from the shared perspective of all parties involved, stakeholder participation and consensus building is a key aspect of defining national indicator sets in the Netherlands. In this article, we shall address the following questions, using patient experiences as an example:

- When and how are stakeholders involved in the development of indicators and instruments that measure the patients' experiences with health care providers?

- Does this involvement lead to indicators and instruments that match stakeholders' information needs?

In other words: is it possible to reach consensus about what needs to be measured and still cover enough of the information needs of various stakeholders to prevent them from developing their own, tailor-made indicator sets? The answer to this question is relevant for researchers and policy makers involved in indicator development.

This article is based on desk research and observations by the authors. It should be noted that the authors are also personally involved in the transparency debate and the development of indicators and instruments namely as director (DD) of the Dutch Centre for Consumer Experience in Health Care ("Centrum Klantervaring Zorg"), as head of the NIVEL research department involved in developing measuring instruments for patient experiences (JR), and as director of NIVEL (PG).

### Indicators in the Dutch health care system

Patient experiences are measured using patient surveys. As part of the policy effort aimed at defining national indicator sets, a standardized method for measuring patient experiences using patient surveys is being promoted by the Ministry of Health, the Inspectorate for Health Care ("Inspectie voor de Gezondheidszorg"), patient/consumer organisations and by insurance companies. This standardized method is called the Consumer Quality Index (CQI). Before describing stakeholder involvement in CQI development, in this section, background information is provided on:

• the reforms in the Dutch health care system;

• the CQI, as a standardized method for measuring patient experiences

• the information needs of stakeholders.

### Regulated competition in the Dutch health care system

In the Dutch health care system, three regulated interdependent markets have been introduced: a health insurance market, a purchasing market, and a health care market. Detailed information and a video about the Dutch health care system and the reforms can be found on the website of the Ministry of Health, Welfare and Sport [10]. Here we shall give a brief overview.

The ***health insurance market ***has been introduced nationwide in 2006 with the introduction of a new insurance system [11,12]. Health insurers offer a uniform, basic benefits package that includes primary care, hospital care, prescription medication, and allied health services such as speech therapy, or physiotherapy for people with chronic diseases. In addition to that, enrolees can take out supplementary insurance, for example for additional physiotherapy (the provision of which is limited under the basic benefits package), dental care, etc. Consumers have free choice of health insurer and can switch once a year. Health insurers are obliged to accept everyone for the basic benefits package. The premium for the basic benefits package must be based on community rating, that is, different health insurers charge different premiums, but health insurers must charge the same premium for all their insured with a specific insurance policy. In other words, insurers are not allowed to differentiate premiums according to the risks of individuals or subgroups. However, they are allowed to offer different insurance policies that may be tuned to the needs of specific subgroups of consumers. The most important difference in insurance policies is between those based on direct payment (guaranteeing the enrolee access to health care providers who are contracted by the health insurer), and those based on restitution (guaranteeing full or partial reimbursement of health care costs incurred by the enrolee).

On the ***purchasing market***, the system aims to provide health insurers with incentives to contract high quality, low-priced health services [13]. This is particularly the case for insurance policies based on direct payment. As a result, it also confronts health insurers with a need for comparative information about the performance of health care providers. They can use this information for selective contracting with preferred providers, and for pay-for-performance contracts aimed at stimulating specific professional behaviour.

On the ***health care market***, consumers with a restitution policy have free choice of providers. Those with a policy based on direct payment can choose from an -usually- comprehensive list of contracted providers. In such a system, patients/consumers too need comparative information about the price and quality of care, in terms of the effectiveness and safety of health services, and patient experiences with access and availability, timeliness, information etc [14].

### Consumer Quality Index

In 2006, the CQI has been proposed by the Ministry of Health, Welfare and Sports as the national standard for measuring patient experiences with health care providers and health plans. CQI is a registered trademark that is owned by the Centre for Consumer Experience in Health Care ("Centrum Klantervaring Zorg"). This Centre is a private foundation with a tripartite board (with members from patient/consumer organisations, health insurers, and health care providers), funded by the Ministry of Health, Welfare and Sports. The CQI trademark is used to certify that information about the performance of health care providers is valid, reliable, and comparable. The trademark indicates that the information has been collected with an official CQI patient survey, by a certified 'survey vendor' according to rules and instructions described in the CQI Manual [15]. The Centre for Consumer Experience in Health Care coordinates the development of CQI surveys and it coordinates data collection with those questionnaires. CQI surveys are developed with both public and private funding. Public funding is used to develop surveys with a high public priority, e.g. in terms of quantity (incidence, prevalence and burden of disease), costs of illness, or in terms of market conditions (based on the extent to which the type of care addressed in the survey is subject to regulated competition). However, data collection is mostly privately funded by health insurers or health care providers who are willing to participate in development projects.

The CQI is based on two principles: the CAHPS [16] method and the QUOTE [17] method. Both instruments measure patient experiences rather than patient satisfaction. Patient experience questionnaires ask whether certain processes and events occurred. In the CQI, this inventory of experiences is combined with questions about values and expectations with regard to health care. For example: patients are asked to report how often in the past 12 months doctors explained things in a way they could understand (never, sometimes, usually or always). In addition, they are asked how important it is to them, that doctors explain things in a way they could understand (on a 4-point scale from 'not important' to 'of the utmost importance'). Combining questions about experiences as well as importance makes it possible to weigh negative experiences. This is helpful in determining priorities for quality improvement by providers. Improvement strategies should target those areas that are very important to patients, but with which they have relatively bad experiences. For informed patient choice, the importance items are irrelevant. Individual patients seeking information about providers or treatments weight experiences of others against their own priorities. Insurance organisations might use information about the importance people attach to aspects of care or service in their purchasing policies.

Currently sixteen CQI questionnaires have been developed for several curative services (such as cataract surgery [18], total hip and total knee arthroplasty [19]), for chronic illnesses (such as diabetes [20]), for health insurance, for general practice, for physiotherapy, for long-term nursing care, and for ambulatory mental health care. In addition to those, another 20 questionnaires are being developed for future use. These include emergency care, care for people with disabilities, inpatient mental health care, veteran health care, cancer care etc. The process of developing a CQI is described in detail in the CQI Manual of the Centre for Consumer Experience in Health Care.

CQI questionnaires are developed in public-private partnerships. The research part of the development project is usually commissioned and funded by public actors, mainly the Ministry of Health, Welfare and Sports and/or the Netherlands organisation for health research and development ("ZonMw"). However, the data collection necessary for the empirical testing of the questionnaires under construction is financed from private sources, i.e. by health care insurers or by health care providers. The data are owned by these private financiers, but the researchers are granted access to the data in order to conduct psychometric and other statistical analyses that are required to validate the instruments. Because large data sets are required to test the discriminative power of CQI instruments, i.e. to test whether an instrument is able to detect differences in the performance of health care providers, the costs of data collection are considerable and generally constitute about half of the total development costs. Total development costs of one CQI instrument can amount up to € 200,000.

### Who wants to know what and why?

In health services research in general, and particularly in areas such as indicator development it is important to define from the start what the purpose of an indicator is. In other words, the question to be kept in mind is "Who wants to know what and why?" If we apply this question to the quality of care of health care providers, different stakeholders have different information needs. As we explained earlier, the performance of health care providers is measured in terms of effectiveness, safety, and patient experiences.

As long as information needs are discussed in such highly abstract terms, parties can quite easily agree on sets of indicators. If stakeholders disagree, they usually do so about the best operationalisation of certain indicators. Take the simple example of an indicator such as "availability of hospital beds". Described as such, this indicator of access and availability may be relevant for several stakeholders, including individual consumers, patient organisations, health insurers, and the Ministry of Health. However, when it comes to operationalising it, the Ministry of Health might ask for the number of beds per 1,000 of the population and perhaps for regional differences in this number. Health insurers may be interested in bed occupancy rates per provider, because they want to know where they can buy additional services. Moreover, for an individual consumer, it is very irrelevant how many beds a certain hospital has. He or she needs only one bed and all that matters is when that bed becomes available.

Also within one group of stakeholders, information needs may differ profoundly. For example, 'the' health care consumer does not exist. People have different preferences and not everyone is keen on making deliberate choices when it comes to health care. Choice is exercised more often by younger, healthier and better-educated patients than by the older, sicker and less well educated [21]. Health literacy and patient activation are important factors that are related to patients' intentions and possibilities to act as an informed consumer [22,23]. However, even among those who do choose, different groups can be identified. Schwartz [24] distinguishes between consumers who are satisficers and maximizers. Satisficers settle for something that is good enough and do not worry about the possibility that there might be a better option. Maximizers seek and accept only the best. It is feasible to assume that the two stereotypes have different information needs. Maximizers are interested in finding out who is the best provider. Satisficers will be more inclined to stick with their usual, local provider but may want to know how this provider performs compared to others. Consumers looking for the 'best provider' may apply different criteria to determine what the 'best' is. Groenewoud [25] for example, found empirical evidence for the existence of two types of consumers: those who focus on outcomes, and those who focus on trust.

Another example of different information needs within one group of stakeholders is between insurance companies who engage in selective contracts and insurance companies who strive for pay-for-performance contracts. Selective contracting requires comparative information, because health insurers want to contract the best and/or the cheapest providers. Pay-for-performance contracts may require information about current performance in relation to external norms or past performance, depending on the type of incentives used [26]. In general, different incentives used call for the application of different statistical techniques, but also for different accents in the measuring instruments used. From the point of view of selective contracting, an indicator on which all providers score equally bad is not interesting. Nevertheless, in a pay-for-performance scheme this indicator might well be the focus of attention, because there is much room for improvement.

The information needs of different stakeholders in the Dutch health care system with regard to the performance of health care providers are summarized in Table 1. The description of information needs in this Table is partly derived from the literature [21-26] and is partly based on the observations of the authors. Considering the differences in information requirements of the various stakeholders, their involvement in indicator development is necessary to make sure that their information needs are met as efficiently and appropriately as possible.

**Table 1:** Information needs of different stakeholders: Who wants to know what?

Who	What
Individual consumers	Maximizers: Who is the best provider for me (in terms of outcomes or in terms of trust)? Where can I find this provider? Do I have access (in terms of waiting times, insurance coverage etc.)? Satisficers: How does my usual provider perform compared to others?

Patient/consumer organisations	Do providers meet quality standards as defined by patient/consumer organisations? Which areas of performance are lacking behind (and therefore need special focus in our lobbying)? How can we help members/patients to make an informed choice?

Health insurers	Do providers meet predefined quality standards (pay-for-performance)? Whom shall we (not) contract from the quality perspective (preferred providers)?

Health care providers	What are best practices? Which areas of our performance need improvement? What do patients and insurers expect from us?

Inspectorate for Health Care	Which providers perform below a minimum quality level (and therefore need further inspection)?

Ministry of Health	What is the overall level of quality of care in the Netherlands and how does it develop over time?

### Stakeholder involvement: when and how

In developmental health services research, such as the development of indicators and the related measuring instruments, it is recommended that researchers and stakeholders meet, debate and cooperate throughout the different phases of a study [27]. Through this process, the results are a co-production. This ensures that information needs are met, which enhances the utilization of the findings. In the development of CQI patient experience questionnaires, there are three phases in which a dialogue between researchers and stakeholders is vital:

- the preparatory phase in which the initial policy problem is transformed or translated into a 'researchable' question;

- the construction phase in which abstract information needs are operationalised in the form of questionnaire items with specific answering categories;

- and the reporting phase in which crude data are being presented in the form of report cards, quality information or policy reports.

**Figure 1:**
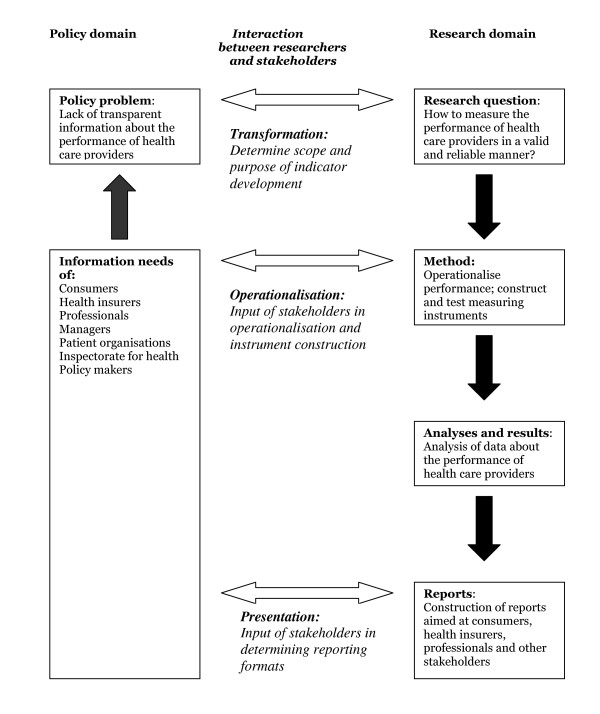
**The research cycle in indicator development (source: adapted from Bensing et al 2001 **[41]).

These phases are visualised in Figure 1. In the remainder of this section, we shall describe the dialogue between researchers and stakeholders in these three phases with respect to the measurement of patient experiences in the Netherlands.

### Preparatory phase (transformation)

Although the publication of comparative information about consumer experiences with health care providers is a vital condition for the functioning of the health care market, the Dutch Ministry of Health, Welfare and Sports has refrained from a central policy concerning the products or providers that should be covered by CQI patient surveys. This is deliberately left to the responsibility of the main actors in health care. The Ministry has mainly determined the process along which decisions about survey development should be taken, namely through tripartite so-called national Steering Committees in which national representatives of patient/consumer organisations, of health care providers and of health insurers should participate. These Steering Committees are installed by the Ministry and are supported by a secretariat resorting under the Inspectorate for Health Care (an autonomous department of the Ministry). The Steering Committees' objectives are to negotiate and implement national agreements per health sector about indicators for external accountability.

Steering Committees have been installed with respect to care for the disabled, long-term nursing care (nursing homes, homes for the elderly, home care), mental health care, hospital care, general practice, physiotherapy and pharmaceutical care. Steering Committees do not only determine which indicators are to be made public by every health care provider, but also how and how often these indicators should be measured. In doing so, Steering Committees can commission and use research on indicator and survey development. CQI patient surveys that have been commissioned by Steering Committees are the CQI Care for the disabled, the CQI Long-term Nursing Care, and the CQI Mental health care.

However, questionnaire development is also initiated by parties outside Steering Committees, particularly in sectors for which Steering Committees have only recently been installed (the CQI pharmaceutical care, the CQI general practice, and the CQI physiotherapy), for chronically ill who use care across various sectors (the CQI Diabetes, the CQI Rheumatoid Arthritis, the CQI Asthma, the CQI COPD), and for particular services that are subject to selective contracting by health insurers (the CQI Cataract, the CQI THA/TKA, the CQI Breast Cancer Care). The initiators of these CQI surveys that are not commissioned by Steering Committees are often health insurers who use the information about patient experiences in their strategic purchasing, but also patient/consumer organisations who need this information for their lobbying and advocacy.

### Construction phase (operationalisation)

CQI surveys are aimed at measuring the quality of care as experienced by health care users. For that reason, the development of every CQI instrument starts with at least two focus group discussions with 8 to 12 patients recruited from the specific target group. Ideally, participants in these focus groups are more or less representative for the target group. In these groups, discussions take place about how patients define good quality of care, and about their concrete experiences with distinct aspects of health care quality. The focus group discussions lead to an operationalisation of quality of care from the patients' perspective and are aimed at ensuring the content validity of the questionnaires.

Typically, the first focus group discussions result in long lists of possible questionnaire items. These long lists contain mostly process aspects of health care quality (e.g. information, communication and interpersonal contact). It is not altogether clear why so few aspects of technical quality and outcomes are mentioned. One of the reasons might be that patients are not necessarily aware of the fact that the technical quality of care varies between doctors and between hospitals. They might take effectiveness and safety of care for granted and, therefore, they are not inclined to mention these aspects explicitly. Furthermore, although they are the ones who *experience *the outcomes of health care interventions, for patients it is difficult to *evaluate *the technical quality of these interventions. For this reason too, technical aspects may not be mentioned in focus group discussions.

In subsequent group discussions, the long lists of items are reduced to short lists that form the basis of the first draft of a questionnaire. Through these qualitative research methods, individual patients are involved in the construction phase of a new CQI questionnaire.

Apart from that involvement, the development of a CQI questionnaire is guided by tripartite working groups meeting 4 to 6 times during the project. If a CQI is developed on the initiative of a Steering Committee, usually, this Steering Committee also installs the working group. In other cases, the Centre for Consumer Experience in Health Care or the research institute responsible for the development of the questionnaire installs the working group.

Members of the working groups are recruited from health insurers, health care providers and patient or consumer organisations. Typically, a working group consists of about ten members: at least two representatives from each party (patient or consumer organisations, providers, and insurers), two or three researchers, and a representative of the Centre for Consumer Experience in Health Care. Working groups are tripartite in the sense that members are recruited from the three parties in health care. However, those parties do not necessarily have equal numbers of representatives in the working group. The number of representatives per party depends on practical issues and on the topic of the CQI questionnaire. If the questionnaire is about one specific type of provider, then the number of providers participating in the working group is relatively small. For example, in the working group for the CQI Physiotherapy there were only two physiotherapists. However, if the questionnaire addresses episodes of care for a certain chronic condition, the number of providers involved is usually much larger. The working group may then include professionals from different disciplines (e.g. general practitioners, specialists, nurses, or allied health professionals).

As a rule, working group members have practical knowledge of the health care sector or the disease under study. For example, the working group for the development of the CQI Rheumatoid Arthritis included:

- the medical advisors of a few large health insurance companies;

- a rheumatologist involved in guideline development within the scientific association of rheumatologists;

- two representatives of the rheumatoid arthritis patient organisation (one staff member of the central bureau of this organisation, and one patient who was active as a volunteer within the organisation).

Although working group members are recruited from insurers, providers and patient organisations, they do not always act as delegates or representatives. They are invited to join the working group for instance because of their involvement in earlier, similar efforts e.g. in the field of indicator development. Particularly medical doctors have often participated in a working group without a formal mandate and feedback from their respective scientific associations.

Generally, the working groups meet at least four times:

- At the start of a development project, when the precise scope of the questionnaire is being discussed and decisions are taken about defining the population, sampling strategy and in-/exclusion criteria. In some cases, an additional meeting is necessary to define the technical details of the sampling strategy.

- Before the first version of the questionnaire is empirically tested, to help researchers with the exact formulation of questions, the translation of complicated medical terms in to lay language etc.

- After the empirical test, to evaluate the outcomes and the reliability and validity of the questionnaire, and to discuss adaptation of the questionnaire in order to improve the psychometric properties and shorten it by discarding superfluous questions, questions with a high item non-response etc.

- At the end of the project, to discuss and approve the research report about the development process, possible other information products (e.g. quality reports or consumer information) and the final version of the questionnaire.

Sometimes, a questionnaire needs to be revised so fundamentally that it takes two extra meetings to address all the changes. This is also the case if parties within a working group have different opinions of the scope and purpose of a questionnaire. In those cases, it takes longer to come to agreement within the working group. This consensus building is crucial in the construction phase of a questionnaire. Working groups do not apply formal methods for consensus building, but rather follow some general principles. The empirical test results serve as guidance in this process. Items that are extremely skewed, have a high item non-response, do not discriminate between health care providers or do not belong to a reliable scale are 'candidates' for deletion. However, working group members may argue to keep some of these items, if they can convince the other parties that these items provide actionable results, are vital to their information need or may become relevant in the near future. In decision about single items, the patient perspective has a certain primacy in the sense that items that result from focus group discussions with patients may not be deleted on the request of health care providers or health insurers.

### Presentation

Whereas in the phase of 'transformation' and 'operationalisation' consensus between the various stakeholders is required, this consensus is not essential when it comes to decisions about the presentation format of CQI data. In the CQI Manuals, three types of information products are described: consumer report cards, purchasing information for insurers and quality reports for health care managers and professionals. These products can be adapted to the information needs of the different stakeholders. In the Netherlands, several studies are currently being conducted around the question how performance information should be presented to various stakeholders in order to best support their decision-making.

The studies with respect to consumer report cards build on earlier work done in the United States by the research group of Hibbard and colleagues [28-31]. Concerning consumer report cards, the Centre for Consumer Experience in Health Care has issued elaborate guidelines for presentation formats. These guidelines are based on the examples of CAHPS consumer reports [32], on studies by Hibbard and on empirical research in the Netherlands, testing various formats in consumer panels [33]. Consumer report cards present information about the relative performance of healthcare providers in the form of stars (* = below average, ** = average, *** = above average). Performance is generally measured on the level of so-called themes (scales consisting of a number of underlying questionnaire items). For consumers who wish to see more in-depth information, additional bar charts can be presented.

As long as parties agree that information is valid and reliable, stakeholders do not necessarily have to agree on the exact content, presentation format and mode of release of CQI data [cf. [34]]. However, consensus is necessary about the timing of publicity. Often, this is decided in Steering Committees. Decisions may be prepared by the CQI working group. However, in sectors where Steering Committees are active, these Committees ultimately decide on the indicators to be measured, the measuring instruments to be used and the frequency of measurement. They also decide on the indicators that are to be made public. However, Steering Committees differ in the degree to which they take responsibility for the presentation format of public performance indicators. For example, the Steering Committee on mental health care has chosen to ensure that data become publicly available, but not to decide on the presentation format of indicators. In contrast, the Steering Committee on long-term nursing care went so far as to determine which indicators were to be made public, on which level of aggregation, and in which format. On the basis of a study [35] commissioned by the Inspectorate for Health Care (one of the participants in the Steering Committee) they chose to present the consumer information in the form of report cards using 1 to 5 stars, representing performance below or far below average to above or far above average. For feedback reports to providers, the Steering Committee chose to present performance scores that reflected actual versus expected performance.

In addition to data collected on the initiative of Steering Committees, health insurers conduct CQI surveys. To that end, they have established a formal cooperation, called the Miletus Foundation ("Stichting Miletus"), in which they pool money for data collection in the developmental phase of an instrument, as well as for data collection with validated questionnaires. The surveys commissioned by the Miletus Foundation usually have nationwide coverage, because all the Dutch health insurers participate in Miletus. That implies that Miletus CQI surveys can be used to construct public consumer information. The Centre for Consumer Experience in Health Care and the Miletus Foundation have formally agreed that:

• Performance data from nationwide Miletus CQI surveys are published as consumer information no later than 6 months after the health insurers have received the internal reports on which they base their contracting decisions;

• Consumer information is prepared according to the guidelines issued in the CQI Manuals.

## Discussion

The questions addressed in this article were: When and how are stakeholders involved in the development of indicators and instruments that measure patient experiences with health care providers? Does this involvement lead to indicators and instruments that match stakeholders' information needs?

With respect to the first question, it can be concluded that the involvement takes the form of tripartite stakeholder representation both on the policy level (Steering Committees) and on the operational level (working groups participating in questionnaire development). With respect to the second question, we argue that this involvement is a prerequisite for collecting information once and using it for multiple purposes. However, a potential risk is that it leads to the development of extremely long questionnaires. We will elaborate on this topic in the next subsection about the operational level. First, we shall address stakeholder involvement on the policy level.

### Policy level

The Steering Committees decide on the indicators to be published, the measuring instruments to be used and the frequency of measurement. Although Steering Committees are assisted by a secretariat and although they have budgets at their disposal for research, advice, instrument development and data processing, there is no legal basis for Steering Committees' decisions. Therefore, the authority of a Steering Committee depends heavily on the mandate of the participants in the committee. This system of obligatory voluntarism works (so far), because the Dutch health care system has a long tradition of central negotiations by national representatives who have a mandate to make binding decisions. However, it is somewhat at odds with a system of regulated competition and progress is often slow, particularly in the curative care sector (hospitals, GPs).

Although Steering Committees are a promising route towards transparency in the Dutch health care system, this strategy has certain inherent disadvantages. One disadvantage is that Steering Committees operate per health care sector, e.g. long-term care, hospital care, or mental health care. The result is that indicators are being developed within the traditional boundaries of health care provision. Moreover, they are being developed first in those sectors where Steering Committees have been installed first, for example in long-term care. These are not necessarily the same areas of health care that matter most from a public health or an economic perspective.

For example, coronary heart diseases, heart failure and stroke are in the Dutch top-10 diseases with the highest burden of disease [36]. Yet, for those patient groups there is no CQI instrument available.

Similarly, the provider-specific CQI questionnaires that are developed on the initiative of Steering Committees do not necessarily cover those areas that are of interest to health insurers. From the point of view of providers, accounting for the quality of care they have delivered in exchange for public funding, the natural unit of analysis is the level of the hospital, or the mental health institution, or the home care provider. However, from the point of view of health insurers, data segmented along the traditional boundaries of health care sectors are often not informative. For example, health insurers can purchase mental health care from hospitals, from mental health institutions, or from self-employed private psychiatrists and psychotherapists. From that point of view, it is much more interesting to know which one of these providers performs best in treating e.g. depression or anxiety disorders. The unit of analysis in that case is not the organisation, but a specific patient group within several organisational settings, and ultimately the performance of an individual medical specialist.

In general, health insurers are interested in comparing the disease-specific or intervention-specific performance of providers, even across the borders of the traditional sectors such as hospital care, mental health care, or primary care. Therefore, health insurers initiate the development of CQI questionnaires in areas that are not taken up by the national Steering Committees. Insurers request the Netherlands organisation for health research and development to fund the development research. Health insurers themselves pay for the data collection through their Miletus Foundation. CQI questionnaires that have been initiated by the Miletus Foundation are, for instance, the CQI Diabetes, the CQI Cataract surgery, the CQI THA/TKA, and the CQI Mobility & Hearing Aides. If health insurers use these questionnaires, they draw samples from their own claims registration (e.g. selecting insured that have been reimbursed for cataract surgery) and organize the data collection, analysis and report without bothering health care providers. Therefore, in that sense, the development, testing and use of these CQI developments does not lead to additional information demands on health care providers. However, health insurers do invite health care providers and patient organisations to their working groups that assist researchers during questionnaire development and testing, thus adding to the burden of representation demands. This overload of representation demands is experienced by the various scientific associations of specialists, but particularly by patient/consumer organisations, whose staff members and volunteers do not only simply lack the time to attend all the meetings, but often also lack the scientific and managerial competences that are essential for fruitful participation [37].

### Operational level

When a CQI instrument is being developed, a working group is installed in which 'representatives' of health insurers, health care providers and patients/consumers participate. It is our observation in these working groups, that representatives of health insurers are often interested in:

- comparatively global information;

- disease-specific or intervention-specific;

- aggregated to the level of contractual partners (e.g. a hospital group, or a chain of diabetes providers);

- on a variety of domains, not only patients' experiences with the quality of the process of care (the items emerging from focus group discussions), but also patients' experiences with the outcome of care ('has your THA/TKA had the expected result?').

Compared to insurers, health care providers want more detailed information on a lower level of aggregation (e.g. the hospital ward instead of the hospital at large). Moreover, contrary to insurers, health care providers usually resent the use of retrospectively measured patient reported outcomes, as invalid measures of the quality of care. In response to this, the Centre for Consumer Experience in Health Care and researchers involved in developing CQI instruments are currently investigating this research domain, looking particularly at recent developments in this area in the English NHS [38]. However, the problem might be conceptual rather than technical. In one CQI development project surgeons argued that the word "quality of care" should consistently be deleted in the research report, because patient experiences -in their opinion- measure something completely different than quality of care. Even after the researchers insisted that the CQI measures quality of care "from the patient's perspective", this remained a topic of debate. The notion that patient experiences are an integral part of the quality of care is apparently not accepted throughout the medical profession.

In general, working group members provide valuable input in all the phases of the process and they are an indispensable source of information for researchers. However, for the acceptation and implementation of a CQI instrument, the fact that working group members are not always official 'representatives' of their group, can be a drawback. The CQI instruments about diabetes, cataract surgery, THA/TKA, breast cancer care, and rheumatoid arthritis have all been developed together with medical doctors, but have not been officially approved or adopted by their scientific associations.

This means that there is a missing link between the operational level and the policy level. At present, this missing link is both a blessing and a curse. It is a blessing, because it makes it possible to continue the development of questionnaires together with specialists, although at the same time on the policy level in the national steering committee, the umbrella organisation of medical specialists is -at best- neutral towards measuring patient experiences and is certainly not one of its frontline advocates.

However, in the end this lack of formal commitment is probably a curse. The whole idea behind introducing a national standard for measuring patient experiences was that we would collect this information once and then use it for multiple purposes. In other words, not only for external accountability, but also for internal quality assurance. The latter use implies that professionals should have a sense of ownership with respect to the CQI instrument. This sense of ownership would improve if the questionnaires were formally adopted by the professional organisations.

Recognition of CQI instruments by the medical profession implies that these professionals accept the notion that patient experiences are an independent but integral part of the quality of care, alongside the technical quality that is captured by indicators of effectiveness and safety. Patient experiences and patient-centred care are not only important in long-term nursing care, where respectful and sensitive interpersonal contact should be core business. This is also important in curative care, where good clinical decision making implies that patients' values and preferences are taken into account and that patients are adequately informed about treatment options and their pros and cons. Partly, the hesitation of medical doctors to acknowledge the value of patient experience surveys stems from their conviction that patients are unable to evaluate the technical quality of care. However, Coulter [39] argues that well designed questionnaires for patients can contribute usefully to an assessment of both the technical competence and interpersonal skills of doctors.

Stakeholder involvement is a prerequisite for collecting information once and using it for multiple purposes. Through stakeholder involvement, we aim to incorporate the information needs of all the potential users of CQI information. However, if those information needs do not fully match, researchers run the risk of developing extremely long questionnaires, that contain all the questions that every stakeholder might possibly at some point in the future be interested in. After all, in comparatively short research projects with hard deadlines, it is easier to add everyone's questions to the questionnaire, than to negotiate with working group members about a core set until consensus has been reached.

This is one of the paradoxes of standardisation. One aims to make one questionnaire that everybody can use, and ends up with something the size of two or more questionnaires. In the CQI, this development is now followed by an effort to make both short and longer versions of the same questionnaire, where the short version can be used for example to make comparative consumer information and the long version can be used for internal quality improvement. However, essentially, this is one-step away from the original purpose.

So, is it possible in performance measurement to reach consensus about what needs to be measured, and still cover enough of the information needs of various stakeholders to prevent them from developing their own, tailor-made indicator sets? Although the Dutch process of developing national indicator sets is still in full swing, we can draw some preliminary conclusions with respect to measuring patient experiences. In our opinion, the sheer number of CQI instruments that has been and still is being developed shows that it is possible to reach consensus about what needs to be measured. Increasingly, CQI instruments are being used instead of a variety of other surveys that were previously used to measure patient experiences or patient satisfaction. Often, this happens to the detriment of the researchers and survey vendors working with these surveys. Preliminary evaluations show that for health care providers and health insurers the benefits of standardization outweigh the possible loss of tailor-made information [40]. However, our observations could be biased by the fact that opponents of the standardized CQI method might not share their views with us. As we stated in the beginning of this paper, we are personally involved -as researchers- in the development of indicators and instruments.

## Conclusion

Consensus about what needs to be measured in performance assessment, is necessary to prevent that health care providers have to deliver data for several sets of indicators, defined by different actors. Consensus can be build through stakeholder involvement in the definition of indicators and measuring instruments.

Our experiences so far show that in this process, particular attention should be given to the participation of medical professionals and of patient/consumer organisations. In the case of medical professionals, a link must be established between the working group member and his or her scientific association. Moreover, in development projects the time schedule must leave room for formal feedback of scientific associations on draft instruments. Currently, research projects are carried out under too many time constraints. In the case of patient/consumer organisations, an extensive programme of support should be implemented, to allow these organisations to not only attend meetings, but to also participate in the scientific discussions and to imprint the "patient's perspective" in every CQI questionnaire.

Stakeholder involvement is complex and time-consuming. However, it is the only way to balance the information needs of all the parties that ask for and benefit from transparency, without frustrating the health care system. The resulting standardization enables contract partners (health care providers and health insurers) to move away from discussions about the validity of indicators and instruments towards discussions about the quality of care.

## Summary

In the Dutch health care system regulated competition has been introduced. In such a system, several actors need comparable information about the performance of health care providers. Without further coordination, these actors will force health care providers to be transparent. For health care providers this might result in a situation in which they have to deliver data for several sets of indicators, defined by different actors: patient or consumer organisations, health insurers, policy makers, or 'watchdogs' such as the Inspectorate for Health Care. To prevent this, an effort is made to define national sets of performance indicators and related measuring instruments. In this article, we have described how are stakeholders involved in the development of indicators and instruments that measure the patients' experiences with health care providers. The question is whether this involvement leads to indicators and instruments that match stakeholders' information needs. The Dutch experiences show that it is possible to implement national indicator sets and to reach consensus about what needs to be measured. Preliminary evaluations show that although stakeholder involvement is complex and time-consuming, the benefits of standardization outweigh the possible loss of tailor-made information.

## Competing interests

The authors declare that they have no competing interests.

## Authors' contributions

DD drafted this manuscript. JR and PP have revised it critically and have given final approval to this version. All authors have read and approved the final manuscript.

## Pre-publication history

The pre-publication history for this paper can be accessed here:

http://www.biomedcentral.com/1472-6963/10/88/prepub
